# Laparoscopic versus open resection of primary colorectal cancers and synchronous liver metastasis: a systematic review and meta-analysis

**DOI:** 10.1007/s00384-023-04375-z

**Published:** 2023-04-05

**Authors:** Stefan Morarasu, Cillian Clancy, Emre Gorgun, Sumeyye Yilmaz, Arpad Ivanecz, Shoji Kawakatsu, Ana Maria Musina, Natalia Velenciuc, Cristian Ene Roata, Gabriel Mihail Dimofte, Sorinel Lunca

**Affiliations:** 1 2nd Department of Surgical Oncology, Regional Institute of Oncology (IRO), Iasi, Romania; 2 Grigore T, Popa University of Medicine and Pharmacy, Iasi, Romania; 3 Department of Colorectal Surgery, Tallaght University Hospital, Dublin 24, Ireland; 4 Department of Colorectal Surgery, Digestive Disease and Surgery Institute, Cleveland Clinic Main Campus, Cleveland, USA; 5 Department of Abdominal and General Surgery, University Medical Center Maribor, Maribor, Slovenia; 6 Division of Surgical Oncology, Department of Surgery, Nagoya University Graduate School of Medicine, Nagoya, Japan

**Keywords:** Laparoscopy, Liver metastases, Colorectal cancer, Surgery, Meta-analysis

## Abstract

**Purpose:**

Combined resection of primary colorectal cancer and associated liver metastases is increasingly common. This study compares peri-operative and oncological outcomes according to surgical approach.

**Methods:**

The study was registered with PROSPERO. A systematic search was performed for all comparative studies describing outcomes in patients that underwent laparoscopic versus open simultaneous resection of colorectal primary tumours and liver metastases. Data was extracted and analysed using a random effects model via Rev Man 5.3

**Results:**

Twenty studies were included with a total of 2168 patients. A laparoscopic approach was performed in 620 patients and an open approach in 872. There was no difference in the groups for BMI (mean difference: 0.04, 95% CI: 0.63–0.70, p = 0.91), number of difficult liver segments (mean difference: 0.64, 95% CI:0.33–1.23, p = 0.18) or major liver resections (mean difference: 0.96, 95% CI: 0.69–1.35, p = 0.83). There were fewer liver lesions per operation in the laparoscopic group (mean difference 0.46, 95% CI: 0.13–0.79, p = 0.007). Laparoscopic surgery was associated with shorter length of stay (p < 0.00001) and less overall postoperative complications (p = 0.0002). There were similar R0 resection rates (p = 0.15) but less disease recurrence in the laparoscopic group (mean difference: 0.57, 95% CI:0.44–0.75, p < 0.0001).

**Conclusion:**

Synchronous laparoscopic resection of primary colorectal cancers and liver metastases is a feasible approach in selected patients and does not demonstrate inferior peri-operative or oncological outcomes.

**Supplementary Information:**

The online version contains supplementary material available at 10.1007/s00384-023-04375-z.

## Introduction

Despite the wide adoption of national screening programs, roughly 25% of patients with colorectal cancer have metastases at diagnosis, with the liver being the most common site of distant spread [1–3]. Patients with colorectal cancer and liver metastasis at diagnosis require a thorough multidisciplinary approach. For patients with resectable colorectal liver metastases (CLM), surgery offers optimal long term survival benefit and possibility of cure. Specialized centres report disease free survival (DFS) rates of 60% at five years after curative resection of the primary and CLM [4–6]. The benefit of a two staged procedure for resection of primary and CLM has been long debated. The recent METASYNC randomized controlled trial (RCT) [7] showed similar perioperative outcomes between synchronous and delayed resection with slightly better overall and disease-free survival in the synchronous group, although these results should be taken with caution given the small sample size. A preference for synchronous resection leaves surgeons with the issue of operative planning as colorectal resections are now widely laparoscopically, whereas the adoption of minimally invasive resection of CLM is not a standard. There is a trend towards a minimally invasive approach for some liver metastases and the OSLO-COMET RCT [8] favoured the use of laparoscopy for parenchymal sparing liver resections as it was associated with fewer postoperative complications, shorter hospital stay and similar negative resection margins. In light of these recent trials and increased support for simultaneous approach of colorectal cancer and CLM, we aimed to perform a systematic review and meta-analysis to compare the surgical outcomes between laparoscopic versus open synchronous resections of colorectal primary and associated liver metastases.

## Materials and methods

### Literature search and study selection

**Fig. 1:**
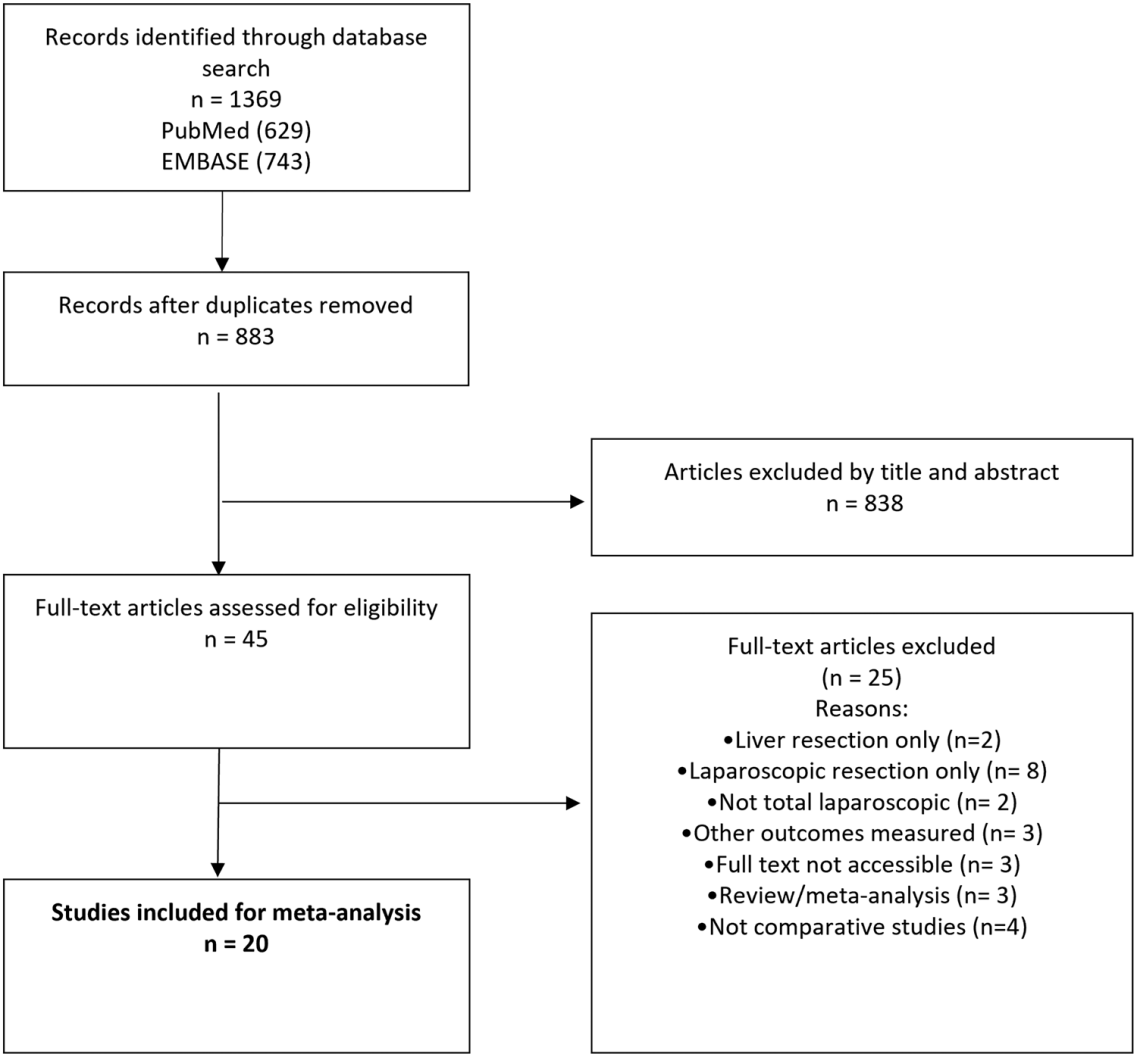
PRISMA flowchart

**Fig. 2:**
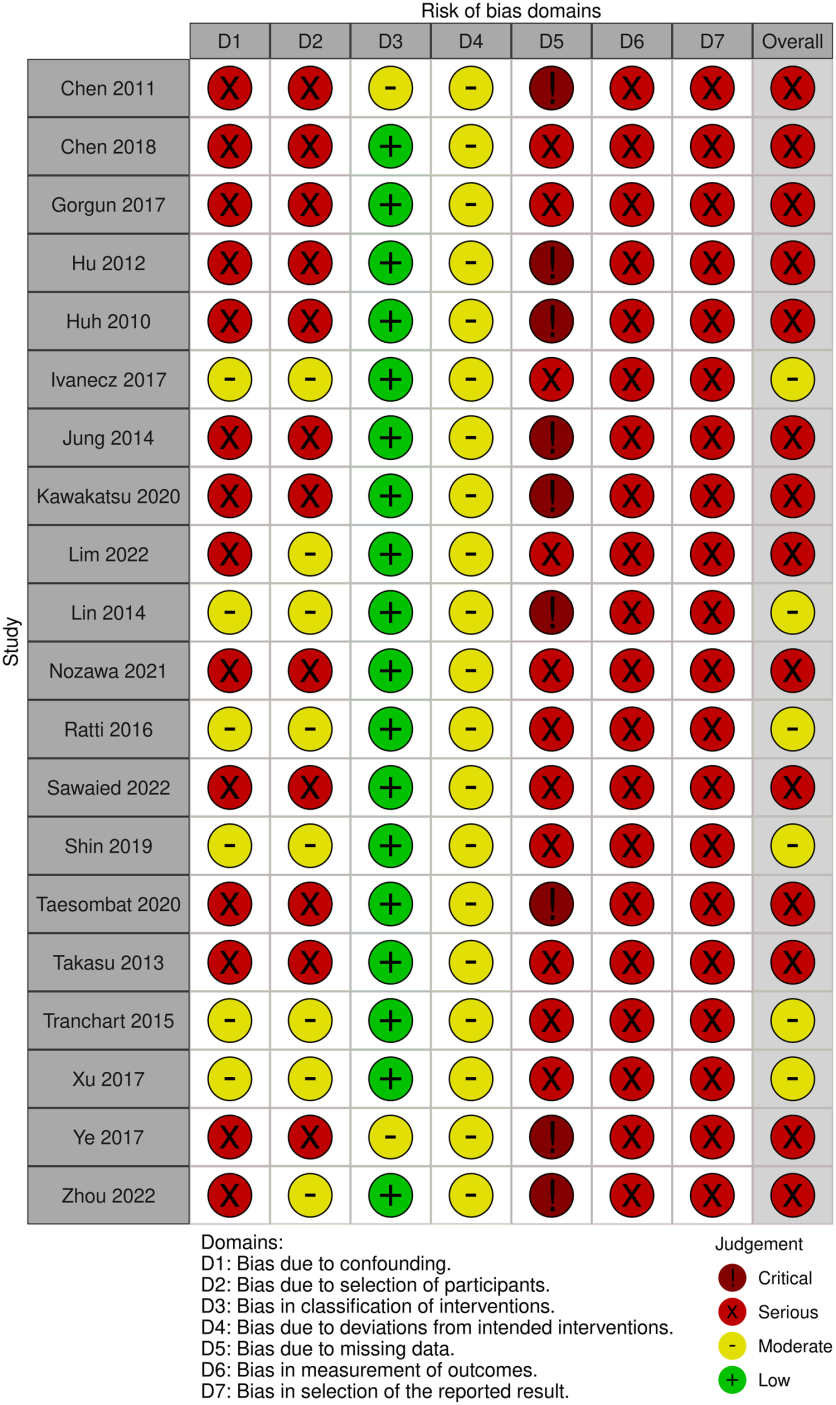
ROBINS-I Risk of bias assessment. Assessment of risk of bias was done by two authors (SM and SL). Each study was classified as low/moderate/serious risk for each of the seven domains. Disagreements were resolved via consensus

The study was registered with PROSPERO (International Prospective Register of Systematic Reviews). The study ID is CRD42022315609. A systematic search of PubMed, EMBASE and SCOPUS databases was performed for all comparative studies examining surgical outcomes in patients that underwent laparoscopic versus open simultaneous resection of colorectal primary tumour and CLM. The following search algorithm was used: (laparoscopic) AND (open) AND (liver OR hepatic) AND (resection). Preferred Reporting Items for Systematic Reviews and Meta-Analyses (PRISMA) guidelines were used as search protocol and the PRISMA checklist was followed to conduct the methodology [9] (Fig. 1). Inclusion criteria were used according to the Problem, Intervention, Comparison and Outcome (PICO) formula. The latest search was performed on February 5^th^, 2023. Two authors (SM and SL) assessed the titles and abstracts of studies found in the search and the full texts of potentially eligible trials were reviewed. Disagreements were resolved by consensus-based discussion. The Newcastle–Ottawa scale (Table 1) and the ROBINS-I tool [10] (Fig. 2) were used to quantify quality of eligible studies. The references of full texts were further screened for additional eligible studies. The corresponding author was contacted to clarify data extraction if additional information was necessary.

**Table 1:** Study characteristics

**First author**	**Year of Publication**	**Type of study**	**Total No. of Patients**	**Laparoscopy No.**	**Open** **No.**	**Age mean**		**Primary Tumour Location**				**NOS**
						**Lap**	**Open**	**Colon**		**Rectum**		
								**Lap**	**Open**	**Lap**	**Open**	
Chen et al. [16]	2011	CM	41	23	18	55	53	NR				7
Chen et al. [17]	2018	CM	38	16	22	66	64.8	4	9	12	12	7
Gorgun et al. [18]	2017	CM	43	14	29	56.3	57.7	6	14	8	15	7
Hu et al. [19]	2012	CM	26	13	13	54	53	7	7	6	6	7
Huh et al. [20]	2010	CM	40	20	20	63	62	7	11	13	9	7
Ivanecz et al. [21]	2017	PSM	20	10	10	62.2	65.4	4	6	6	4	8
Jung et al. [22]	2014	CM	48	24	24	60	60	18	16	6	8	7
Kawakatsu et al. [23]	2020	CM	141	37	104	65	64.5	13	24	58	43	7
Lim et al. [24]	2022	PSM	647	48	136	61	62	30	89	18	47	7
Lin et al. [25]	2014	PSM	72	36	36	57.5	57.4	18	19	18	17	7
Nozawa et al. [26]	2021	CM	53	17	36	64	68	10	22	7	14	7
Ratti et al. [27]	2016	PSM	75	25	50	60	62	13	27	12	23	7
Sawaied et al. [28]	2022	CM	63	21	42	61	64	17	32	4	9	7
Shin et al. [29]	2019	PSM	218	109	109	56	59	77	81	32	38	8
Taesombat et al. [30]	2020	CM	36	12	24	69.4	63.3	8	11	4	13	8
Takasu et al. [31]	2013	CM	14	7	7	74	62	3	3	4	4	6
Tranchart et al. [32]	2015	PSM	178	89	89	66.6	65	38	51	41	38	8
Xu et al. [33]	2017	PSM	54	25	29	58.2	59.6	15	12	5	5	7
Ye et al. [34]	2017	CM	80	40	40	62.5	62.3	27	26	13	14	7
Zhou et al. [35]	2022	PSM	281	34	34	59.4	58.6	10	8	24	26	7

*CM* case-match, *PSM* propensity score matched, *NOS* Newcastle–Ottawa scale

### Eligibility criteria

Studies written in English including comparative data between laparoscopic versus open synchronous colorectal and CLM resection were assessed for eligibility. The primary end points were intraoperative blood loss, operative time, length of stay and overall postoperative morbidity, which were also compared in subgroups based on primary tumour location. Secondary end points were rate of R0 resections and disease recurrence. Studies without comparative data were not included. Studies in which resection of primary and liver metastases was not done by a minimally invasive approach were excluded.

### Data extraction and outcomes

For each eligible study the following data was recorded: author’s names, journal, year of publication, study type, total number of patients and number of patients included in each group, primary tumour location, mean age, preoperative risk factors for increased surgical difficulty (BMI, lesions in difficult liver segments, mean number of liver lesions, liver metastasis maximum mean size, major liver resections), operative outcomes (operative time, blood loss, length of stay) and postoperative outcomes (postoperative complications, overall morbidity, R0 resections and recurrence free survival). The type of procedure was recorded and defined as: synchronous laparoscopic colorectal and liver resection (LAP group) and synchronous laparoscopic/open colorectal resection and open liver resection (OPEN group). The two groups were compared in a meta-analytical model based on three blocks of variables: i) risk factors for increased surgical difficulty (BMI, number of difficult liver segments – 1,4a,7,8, mean number of liver lesions, liver lesion maximum mean size, major liver resections); ii) operative outcomes (operative time, blood loss, length of stay); iii) oncological outcomes (overall morbidity, R0 resections, disease recurrence).

### Subgroup analysis

To enable subgroup analysis of operative outcomes based on location of primary tumour (rectum versus left colon versus right colon) authors were asked to provide individual patient data, unpublished in the original studies. Three subgroups of patients were formed (rectum, left colon and right colon) and were compared in terms of operative outcomes only (i.e., operative time, intraoperative blood loss, length of stay and morbidity).

### Statistical analysis

Random-effects models were used to measure all pooled outcomes as described by Der Simonian and Laird [11] and the odds ratio (OR) was estimated with its variance and 95% confidence interval (CI) for dichotomous variables while mean difference (MD) 95% CI was used for continuous data. The random effects analysis weighted the natural logarithm of each study's OR by the inverse of its variance plus an estimate of the between-study variance in the presence of between-study heterogeneity. As described previously [12–14], heterogeneity between ORs for the same outcome between different studies was assessed using the I [2] inconsistency test and chi-square-based Cochran’s Q statistic test [15] in which p < 0.05 is taken to indicate the presence of significant heterogeneity. Analyses were conducted using Review Manager 5.3.

## Results

### Eligible studies

Twenty studies [16–35] containing data comparing laparoscopic versus open synchronous resection of primary and CLM were included (Table 1). The initial search found 1369 studies. After excluding duplicates and unrelated studies based on abstract triage, 45 full texts were assessed for eligibility, out of which 20 matched the inclusion criteria and were analysed. Year of publication of included studies ranged from 2010 to 2022. All studies were case matched. Eight studies were propensity-matched [21, 24–27, 30, 31, 35]. The total number of included patients was 1492, split into two groups: study group (LAP, n = 620) and control group (OPEN, n = 872). Mean age in the LAP group was 61.6 ± 5 vs 61.3 ± 3.75 in the OPEN group. Mean BMI was 23.29 ± 1.75 in both groups. Location of primary tumour (e.g., colon, rectum) was comparable between the two groups with 53.5% (n = 335) colon tumours and 46.5% (n = 291) rectal tumours in the LAP group versus 58.2% (n = 468) colon tumours and 41.8% (n = 335) rectal tumours in the OPEN group. Three studies [18, 21, 23] provided raw data for subgroup comparison of operative outcomes (Table 2).

**Table 2:** Subgroup analysis of operative outcomes based on primary tumour location (rectum vs left colon vs right colon)

		**Gorgun et al.** [18]			**Ivanecz et al.** [21]			**Kawakatsu et al.** [23]		
		**Location of primary tumour**			**Location of primary tumour**			**Location of primary tumour**		
		**Rectum**	**Left**	**Right**	**Rectum**	**Left**	**Right**	**Rectum**	**Left**	**Right**
**No of patients**	**Open**	15	5	9	4	3	3	43	37	24
	**Lap**	8	1	5	5	2	2	24	9	4
**Operative time** **(minutes ± SD)**	**Open**	348.8 ± 44.6	350.2 ± 44.8	321 ± 54.5	302.5 ± 30	250 ± 30	203 ± 27.5	639 ± 173.2	589.8 ± 138.9	447.2 ± 101.5
	**Lap**	367 ± 56.7	N/A	272.4 ± 47.9	320 ± 30	155 ± 2.5	170 ± 5	553.6 ± 160.1	423 ± 82.3	506 ± 71.1
**Blood loss** **(mL ± SD)**	**Open**	675.3 ± 166.2	500 ± 168.1	459.4 ± 243.9	307.5 ± 172.9	136.6 ± 47.8	80 ± 72.5	574.3 ± 388.1	659.1 ± 490.6	764.6 ± 425.7
	**Lap**	380 ± 48.2	N/A	290 ± 68.3	198 ± 129.2	20 ± 20	15 ± 15	252 ± 193.3	108.3 ± 124.8	326.2 ± 232.7
**Length of stay** **(days ± SD)**	**Open**	10.8 ± 1.3	11.2 ± 3.0	8.1 ± 0.7	28 ± 18.1	11 ± 0.8	17 ± 11.3	22.1 ± 12.7	17.4 ± 6.6	18.9 ± 12.3
	**Lap**	6.6 ± 1.4	N/A	6.2 ± 0.8	15.6 ± 13.3	7.5 ± 0.5	8	17.2 ± 8.1	10.8 ± 1.9	15.5 ± 4
**Morbidity**	**Open**	7	2	4	2	0	1	7	0	3
	**Lap**	1	0	0	2	0	0	1	0	1

### Preoperative risk factors for increased surgical difficulty

#### BMI

**Fig. 3:**
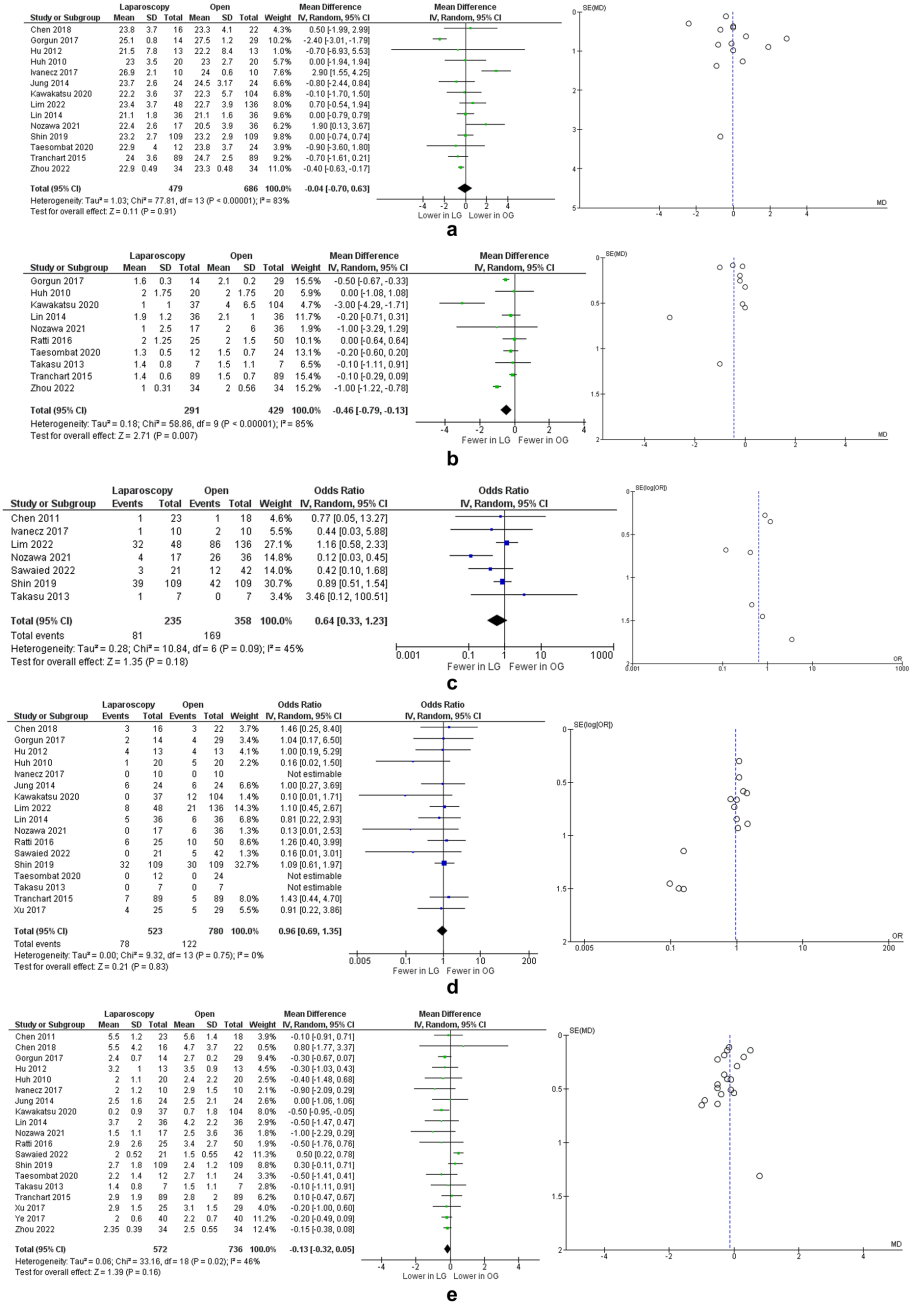
Meta analysis of preoperative risk factors for increased surgical difficulty: (**a**) BMI; (**b**) mean number of liver lesions; (**c**) difficult segments; (**d**) major liver resections; (**e**) colorectal liver metastasis mean size. **Legend:** Each study is shown by the point estimate of the odds ratio/mean difference (OR/MD; square proportional to the weight of each study) and 95% confidence interval (CI) for the OR (extending lines); the combined ORs/mean difference and 95% CIs by random effects calculations are shown by diamonds. **a** LAP versus OPEN and BMI (n = 1165, p = 0.91; test for heterogeneity Cochran Q: 77.81, df: 13, p < 0.00001, I^2^: 83%) **b** LAP versus OPEN and mean number of liver lesions (n = 720, p = 0.007; test for heterogeneity Cochran Q: 58.86, df: 9, p < 0.00001, I^2^: 85%) **c** LAP versus OPEN and difficult segments (n = 593, p = 0.18; test for heterogeneity Cochran Q: 10.84, df: 6, p = 0.09, I^2^: 45%) **d** LAP versus OPEN and major liver resections (n = 1303, p = 0.83; test for heterogeneity Cochran Q: 9.32, df: 13, p = 0.75, I^2^: 0%) **e** LAP versus OPEN and colorectal liver metastasis mean size (n = 1308, p = 0.16; test for heterogeneity Cochran Q: 33.16, df: 18, p = 0.02, I^2^: 46%)

Fourteen studies [17–28, 30, 35] describing 1165 patients included data on patients BMI. There was no difference in BMI between the 2 groups (mean difference: 0.04, 95% CI: [0.63, 0.70], p = 0.91, Chi^2^ = 77.81, I^2^ = 83%) (Fig. 3A).

#### Number of liver lesions

Ten studies [18, 19, 23–26, 28–30, 35] including 720 patients reported the mean number of liver lesions for each group. There were statistically fewer lesions in the LAP group with a mean difference of 0.46, 95% CI: [0.13, 0.79], p = 0.007, Chi^2^ = 58.86, I^2^ = 85% (Fig. 3B).

#### Difficult segments

Seven studies [16, 21, 24, 25, 27–29] provided data on presence of liver lesions in surgically difficult liver segments (segments 1, 4a, 7 and 8). There was no significant difference between the two groups (OR: 0.64, 95% CI: [0.33,1.23], p = 0.18, Chi^2^ = 10.84, I^2^ = 45%) (Fig. 3C).

#### Major liver resections

Seventeen studies [17–33] had data on the number of major liver resections in each group. There was no significant difference between LAP and OPEN group (OR: 0.96, 95% CI: [0.69,1.35], p = 0.83, Chi^2^ = 9.32, I^2^ = 0%) (Fig. 3D).

#### Colorectal liver metastasis mean size

Nineteen studies [16–33, 35] reported the mean diameter of the largest liver lesion in both groups. There was no difference between the two groups (mean difference: 0.13 cm, 95% CI: [0.05, 0.32], p = 0.16, Chi^2^ = 33.16, I^2^ = 46%) (Fig. 3E).

### Operative outcomes

#### Operative time

**Fig. 4:**
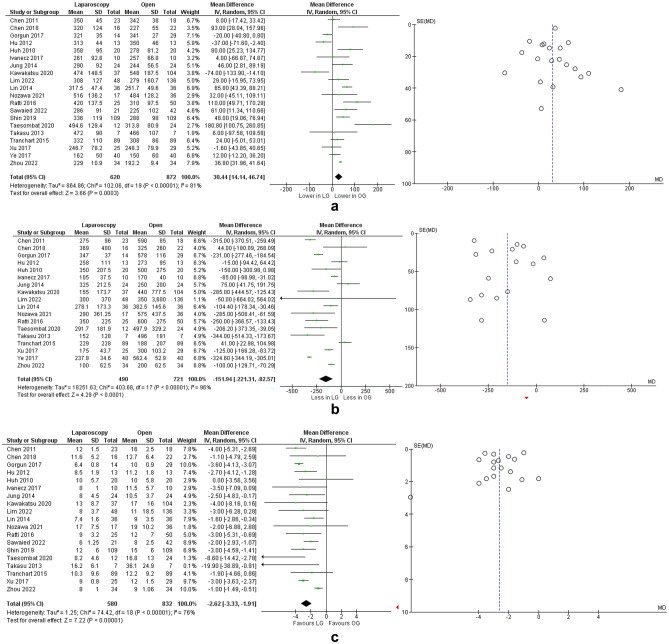
Meta analysis of operative outcomes: (**a**) operative time; (**b**) blood loss; (**c**) length of stay **Legend:** Each study is shown by the point estimate of the odds ratio/mean difference (OR/MD; square proportional to the weight of each study) and 95% confidence interval (CI) for the OR (extending lines); the combined ORs/mean difference and 95% CIs by random effects calculations are shown by diamonds. **a** LAP versus OPEN and operative time (n = 1492, p = 0.0003; test for heterogeneity Cochran Q: 102.06, df: 19, p < 0.00001, I^2^: 81%) **b** LAP versus OPEN and intraoperative blood loss (n = 1211, p < 0.0001; test for heterogeneity Cochran Q: 403.68, df: 17, p < 0.00001, I^2^: 96%) **c** LAP versus OPEN and length of hospital stay (n = 1412, p < 0.00001; test for heterogeneity Cochran Q: 74.42, df: 18, p < 0.00001, I^2^: 76%)

All studies [16–35] provided data on operative time. The operative time was significantly lower in the OPEN group (mean difference: 30.44 min, 95% CI: [14.14, 46.74], p = 0.0003, Chi^2^ = 102.06, I^2^ = 81%) (Fig. 4A). Three studies [18, 21, 23] provided new data to enable subgroup analysis of operative time according to tumour location (Table 2). For lesions located in the rectum, there was no significant difference in terms of operative time (mean difference: 4.61 min, 95% CI: [44.79,54.01], p = 0.85, Chi^2^ = 5.34, I^2^ = 63%) (Suppl. Fig. 6A). Operative time was shorter for left colectomies in the LAP group, however only two studies [21, 23] had enough data for comparison (mean difference: 124.14 min, 95% CI: [55.04, 193.24], p = 0.0004, Chi^2^ = 3.27, I^2^ = 69%) (Suppl. Fig. 7A). In the right colon subgroup, the operative time was comparable (mean difference: 17.75 min, 95% CI: [31.17, 66.66], p = 0.48, Chi^2^ = 5.08, I^2^ = 61%) (Suppl. Fig. 8A).

#### Blood loss

Eighteen studies [16–32, 35] measured the operative blood loss. There was significantly less blood loss in the LAP group (mean difference: 151.94 mL, 95% CI: [82.57, 221.31], p < 0.0001, Chi^2^ = 403.68, I^2^ = 96%) (Fig. 4B). Even in subgroup analysis, less blood loss was seen in the LAP group regardless of primary cancer location: i) rectum subgroup, mean difference: 269.90 mL, 95% CI: [172.07, 367.73], p < 0.00001, Chi^2^ = 3.15, I^2^ = 37% (Suppl. Fig. 6B); ii) left colon subgroup, mean difference: 325.32 mL, 95% CI: [99.87, 750.51], p = 0.13, Chi^2^ = 20.50, I^2^ = 95% (Suppl. Fig. 7B); iii) right colon subgroup, mean difference: 181.03 mL, 95% CI: [5, 357.06], p = 0.04, Chi^2^ = 6.67, I^2^ = 70% (Suppl. Fig. 8B).

#### Length of stay

Nineteen studies [16–33, 35] reported on the length of hospital stay among the two groups. LAP patients were discharged significantly earlier compared to OPEN patients (mean difference: 2.62 days, 95% CI: [1.91, 3.33], p < 0.00001, Chi^2^ = 74.42, I^2^ = 76%) (Fig. 4C). When subgroups were analysed, results remained in favour of LAP group, regardless of primary cancer location: i) rectum subgroup, (mean difference: 4.26 days, 95% CI: [3.12, 5.40], p < 0.00001, Chi^2^ = 0.64, I^2^ = 0%) (Suppl. Fig. 6C); ii) left colon subgroup, (mean difference: 4.85 days, 95% CI: [1.84, 7.86], p = 0.002, Chi^2^ = 5.01, I^2^ = 80%) (Suppl. Fig. 7C); iii) right colon subgroup, (mean difference: 1.93 days, 95% CI: [1.10, 2.76], p < 0.00001, Chi^2^ = 0.21, I^2^ = 0%) (Suppl. Fig. 8C).

### Postoperative outcomes

#### Overall morbidity

**Fig. 5:**
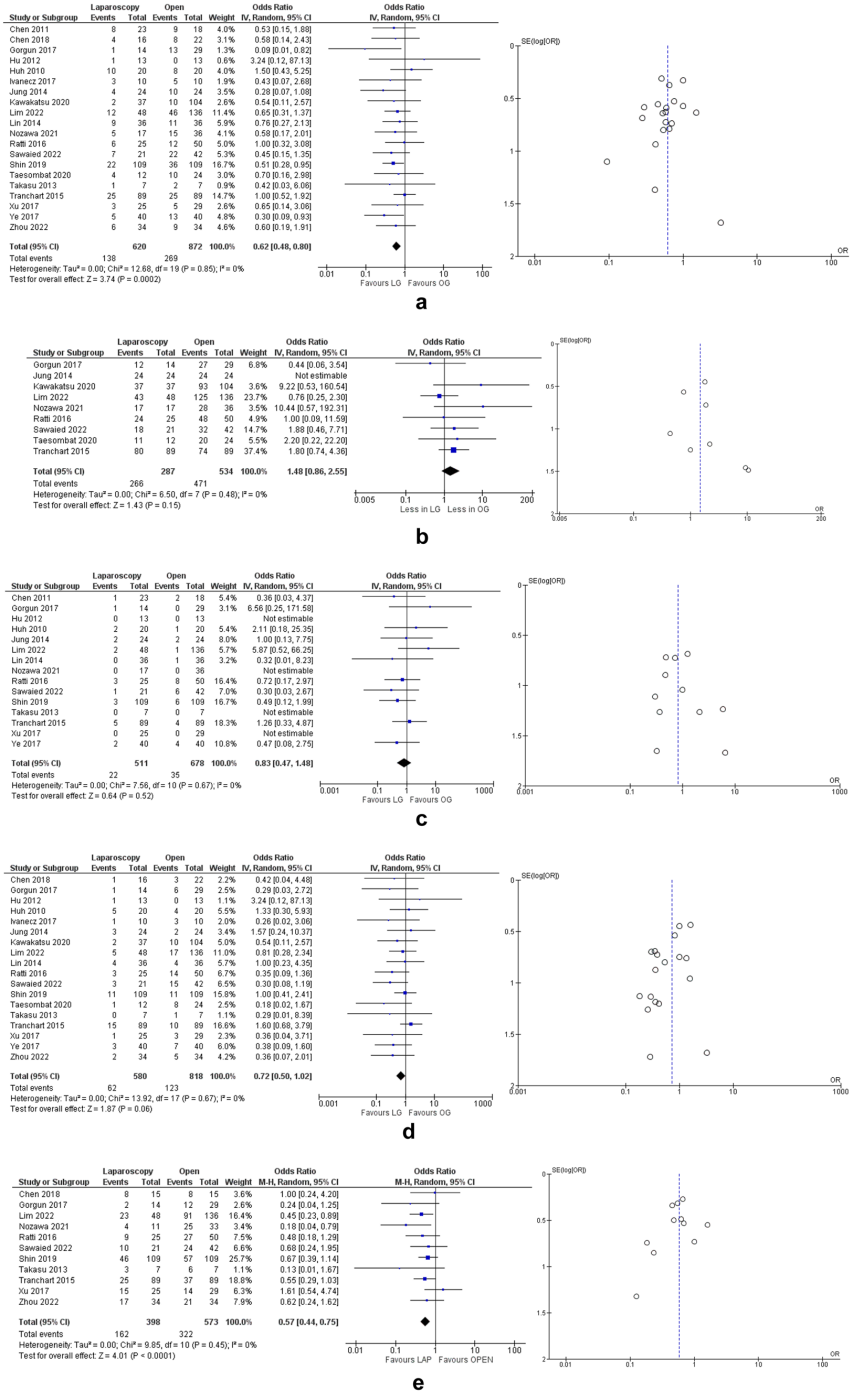
Meta analysis of postoperative outcomes: (**a**) overall morbidity; (**b**) liver R0 resections; (**c**) anastomotic leaks; (**d**) major complications; (**e**) disease recurrences. **Legend:** Each study is shown by the point estimate of the odds ratio/mean difference (OR/MD; square proportional to the weight of each study) and 95% confidence interval (CI) for the OR (extending lines); the combined ORs/mean difference and 95% CIs by random effects calculations are shown by diamonds. **a** LAP versus OPEN and overall morbidity (n = 1492, p = 0.0002; test for heterogeneity Cochran Q: 12.68, df: 19, p = 0.85, I^2^: 0%) **b** LAP versus OPEN and liver R0 resections (n = 821, p = 0.15; test for heterogeneity Cochran Q: 6.50, df: 7, p = 0.48, I^2^: 0%) **c** LAP versus OPEN and anastomostic leaks (n = 1189, p = 0.52; test for heterogeneity Cochran Q: 7.56, df: 10, p = 0.67, I^2^: 0%) **d** LAP versus OPEN and major complications defined as Clavien-Dindo III and IV (n = 1398, p = 0.06; test for heterogeneity Cochran Q: 13.92, df: 17, p = 0.67, I^2^: 0%) **e** LAP versus OPEN and disease recurrences (n = 971, p < 0.0001; test for heterogeneity Cochran Q: 9.85, df: 10, p = 0.45, I^2^: 0%)

All studies [16–35] had data on postoperative morbidity. LAP group had significantly fewer complications (OR: 0.62, 95% CI: [0.48, 0.80], p = 0.0002, Chi^2^ = 12.68, I^2^ = 0%) (Fig. 5A). Subgroup analysis was possible only for primaries located in the rectum and right colon from three studies which provided data (Table 2). When analysed separately, both subgroups showed similar morbidity regardless of operative approach: i) rectum subgroup, OR: 0.27, 95% CI: [0.07, 1.04], p = 0.06, Chi^2^ = 12.33, I^2^ = 0% (Fig. 6D); ii) right colon subgroup, OR: 0.56, 95% CI: [0.09, 3.68], p = 0.55, Chi^2^ = 2.27, I^2^ = 12% (Fig. 8D). Fifteen studies reported 30-day mortality in the two grups. Meta-analysis was not performed as there were very few cases: out of 1212 patients (490 in the LAP group and 722 in the OPEN group) there were five postoperative deaths (two in the LAP group and three in the OPEN group).

#### R0 resections

Nine studies [18, 22–26, 28–30] analysed the number of R0 liver resections in each group. There was no significant difference and heterogeneity in terms of R0 resections (OR: 1.48, 95% CI: [0.86, 2.55], p = 0.15, Chi^2^ = 6.50, I^2^ = 0%) (Fig. 5B).

#### Anastomotic leaks

Fifteen studies [16, 18–20, 22, 24–29] analysed the number of colorectal anastomotic leaks between the two main groups. There was no significant difference in terms of leaks (OR: 0.83, 95% CI: [0.47, 1.48], p = 0.52, Chi^2^ = 7.56, I^2^ = 0%) (Fig. 5C).

#### Major complications

Eighteen studies [17–25, 27–35] reported on the incidence of major complications (Clavien Dindo III and IV). There was no significant difference in terms of major complications between LAP and OPEN group (OR: 0.72, 95% CI: [0.50, 1.02], p = 0.06, Chi^2^ = 13.92, I^2^ = 0%) (Fig. 5D).

#### Disease recurrence

Eleven studies [17, 18, 24–31, 35] had descriptive, numerical data on the number of recurrences in each group at a three-year interval. Six studies [17, 18, 26, 28, 29, 35] specified the site of recurrence, with liver being the most common location. The forest plot showed significantly fewer overall recurrences in the LAP group (OR: 0.57, 95% CI: [0.44, 0.75], p < 0.0001, Chi^2^ = 9.85, I^2^ = 0%) (Fig. 5E).

## Discussion

Our meta-analysis showed that laparoscopic synchronous resection of primary colorectal tumour and associated liver metastases can be performed safely, retaining the widely known operative advantages of minimally invasive surgery when compared to open surgery (e.g., less blood loss, shorter length of stay and improved morbidity), although LAP group is associated with longer operative times (mean difference: 29.98 min). These results are endorsed by preoperative similarities between the two groups of patients in terms of surgical difficulty (e.g., comparable BMI, number of difficult segments and number of major liver resections, mean size of colorectal liver metastasis), except for the number of liver lesions. In the laparoscopy group there were fewer liver lesions per patient, although this difference was small (mean difference 0.46). The significance of preoperative similarities in the two groups is limited due to lack and discrepancy of data; while most studies had data on major liver resections or mean size of liver metastasis, few of them (n = 7) provided data on the presence of metastasis in difficult segments for each group. This might imply that laparoscopy was chosen for fewer and more accessible lesions. Given there were fewer metastases in the laparoscopic group it may be the case that selection bias exists regarding patients undergoing laparoscopic surgery however the findings here support that where possible laparoscopic surgery is a good option with shorter stays and equivalent R0 resection rates, although not all studies (n = 9) reported on the R0 resection rate. This is crucial when analysing oncological outcomes and future studies should ensure that rate of negative margins is reported in each group.

The feasibility of laparoscopic versus open liver resection is already proven in the OSLO-COMET trial [8], however synchronous laparoscopic resection of liver metastases and the primary colorectal tumour is yet to be analysed in a trial. All current comparative studies favour the use of laparoscopy for a combined approach and our analysis agrees with individual studies. The combined approach is associated with increased morbidity compared to a staged resection due to longer operative time, surgical trauma, long or multiple incisions, however the majority of patients in the combined group were operated at least partially open, usually the liver [36, 37]. With a total laparoscopic approach the surgical trauma is significantly reduced and, as per the aforementioned results, it improves morbidity, bearing in mind that centre experience in both colorectal and liver minimally invasive surgery is imperative. We must also add that, overall, morbidity was better in the laparoscopy group, however when considering only major complications (Clavien-Dindo III and IV) the two groups were comparable, suggesting that the more frequent complications in the OPEN group are usually minor ones (Clavien-Dindo I and II) likely related to the laparotomy. A network meta-analysis published in 2015 showed no difference in postoperative outcomes when the three approaches were compared: combined versus liver first versus colorectal first [38]. This highlights that a combined approach is feasible in selected patients as it decreases the overall hospital stay and favours a quicker start of systemic treatment if the patients are surgically recovered in similar time compared to single resections (liver or colorectal). More so, as per the recent iCral study [39], a combined resection does not increase the risk of anastomotic leaks. Our meta-analysis confirms this and adds that the addition of laparoscopy does not increase the leak rate. Although a total laparoscopic approach is associated with a longer operative time, a difference of roughly thirty minutes should not be clinically relevant for major abdominal operations.

When performing subgroup analysis based on location of primary tumour, laparoscopy did not show inferior operative outcomes, however only three studies responded and provided raw data enabling subgroup analysis, thus results on subgroup comparison are limited due to the small sample size. Further studies should analyse operative outcomes also based on primary cancer location as it influences surgeons` planning of incision length, type and numbers of ports. Performing a laparoscopic right colectomy combined with an open liver resection is somewhat counterintuitive as an open right colectomy can be performed through the same incision, whereas a laparoscopic/robotic anterior resection combined with an open hepatectomy is feasible to reduce length of incision and surgical trauma.

Our results showed that synchronous laparoscopic resection is associated with improved recurrence free survival at three years compared to open surgery. This contradicts individual studies which showed similar recurrence rates between the two groups. The number of liver lesions was indeed significantly smaller in the laparoscopic group, which could partially explain the improved recurrence rate by reflecting a more advanced stage of systemic disease [40] despite the R0 resection rates being similar. It is likely there is selection bias present. Regardless, a pragmatic approach would be to perform laparoscopic combined resection where technically possible. More so, the recovery after major open surgery is slower and might lead to delayed initiation of adjuvant chemotherapy. This might have influenced the lower recurrence rate in the laparoscopic group.

This is the most up to date meta-analysis on synchronous laparoscopic versus open resection of primary and CLM and the first to analyse the two groups in terms of risk factors for increased surgical difficulty in addition to operative and postoperative outcomes. In agreement with Pan et al.’s study [41] and previously published systematic reviews [42, 43] laparoscopic approach showed fewer complications, less blood loss and shorter hospital stay. In addition, our study showed that the two groups were comparable in terms of surgical selection criteria, except number of liver lesions, where fewer lesions were found in the laparoscopic group, although we must highlight significant inter-study variability especially when the two groups were compared in terms of selection criteria, suggesting a probability of selection bias or simply that the cohorts in each study were significantly different in terms of preoperative variables. Similarly, when looking at the operative outcomes such as operative time, blood loss and length of stay there was significant variability in the collected data with I^2^ ranging from 76 to 96%. However, the variability herein should be interpreted with caution as the outcomes here depend a lot on the operator itself (for operative time and blood loss) and on each hospital practice when considering length of stay. In contrary, when analysing postoperative outcomes such as R0 resections, recurrences or complications which can be quantified in an objective and standardized manner, the data was homogenous with I^2^ estimates of 0% in all comparisons.

Performing a randomized controlled trial would not be pragmatic as the heterogeneity of liver lesion location combined with complexity of colorectal resection must always be considered. Where it is possible to perform minimally invasive surgery the laparoscopic approach is associated with better peri-operative outcomes and at least equivalent oncological outcomes.

## Conclusion

The building body of evidence endorses a synchronous laparoscopic approach for combined resection of primary tumour and associated colorectal liver metastases. Laparoscopy is associated with longer operative times, but with less blood loss, shorter length of stay, fewer postoperative complications and similar R0 resection rates. Interestingly, laparoscopy was associated with fewer overall recurrences. Further clinical trials should address this and reiterate current results.

### Supplementary Information

Below is the link to the electronic supplementary material.

Supplementary file1 (DOCX 710 KB)

Supplementary file2 (DOCX 1182 KB)

Supplementary file3 (DOCX 622 KB)

## Data Availability

The data that support the findings of this study are available on request from the corresponding author, CC.
