# Supplementary material for: Laparoscopic versus open resection of primary colorectal cancers and synchronous liver metastasis: a systematic review and meta-analysis
Source: Int J Colorectal Dis. 2023 Apr 5;38(1):90. doi: 10.1007/s00384-023-04375-z (PMC10076361; doi:10.1007/s00384-023-04375-z)
Supplement: Supplementary file 1 — Supplementary file1 (DOCX 710 KB) [file 384_2023_4375_MOESM1_ESM.docx]

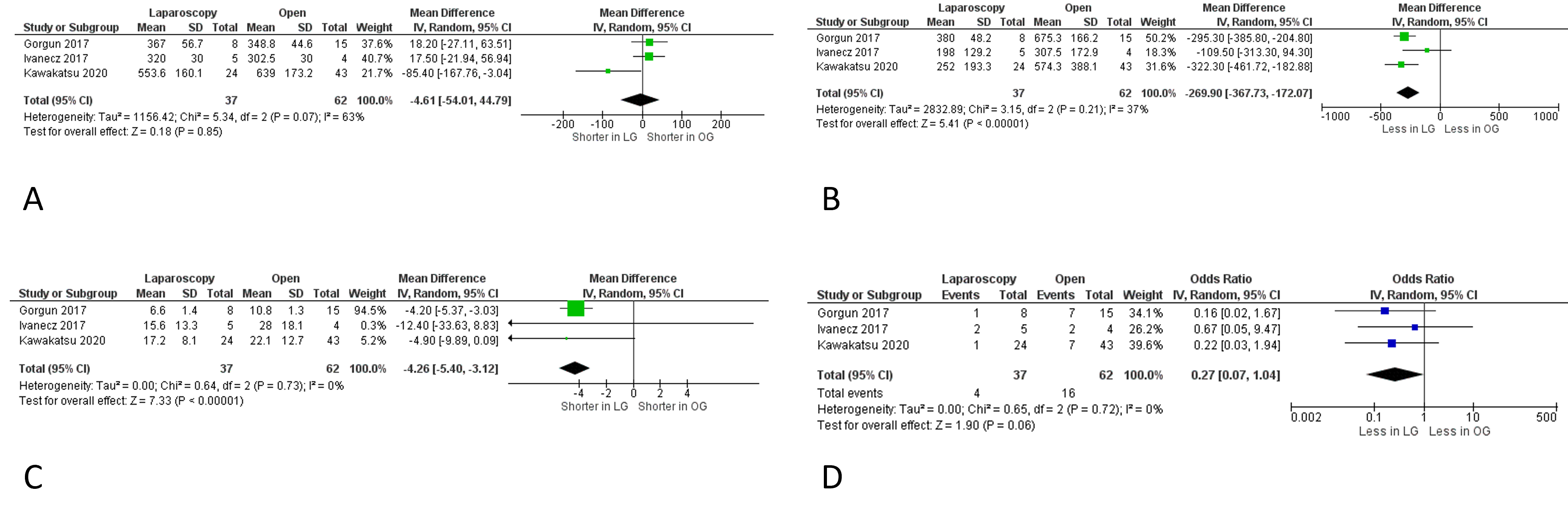


**Supplementary Fig 6:** Subgroup meta-analysis of operative outcomes in patients with primary tumour located in the rectum: (a) operative time; (b) intraoperative blood loss; (c) length of stay; (d) morbidity

**Legend:** Each study is shown by the point estimate of the odds ratio/mean difference (OR/MD; square proportional to the weight of each study) and 95% confidence interval (CI) for the OR (extending lines); the combined ORs/mean difference and 95% CIs by random effects calculations are shown by diamonds.

(a) Rectum only; LAP versus OPEN and operative time (n=99, p=0.85; test for heterogeneity Cochran Q: 5.34, df: 2, p=0.07, I^2^: 63%)

(b) Rectum only; LAP versus OPEN and blood loss (n=99, p<0.00001; test for heterogeneity Cochran Q: 3.15, df: 2, p=0.21, I^2^: 37%)

(c) Rectum only; LAP versus OPEN and length of stay (n=99, p<0.00001; test for heterogeneity Cochran Q: 0.64, df: 2, p=0.73, I^2^: 0%)

(d) Rectum only; LAP versus OPEN and morbidity (n=99, p=0.06; test for heterogeneity Cochran Q: 0.65, df: 2, p=0.72, I^2^: 0%)
