# Supplementary material for: Laparoscopic versus open resection of primary colorectal cancers and synchronous liver metastasis: a systematic review and meta-analysis
Source: Int J Colorectal Dis. 2023 Apr 5;38(1):90. doi: 10.1007/s00384-023-04375-z (PMC10076361; doi:10.1007/s00384-023-04375-z)
Supplement: Supplementary file 2 — Supplementary file2 (DOCX 1182 KB) [file 384_2023_4375_MOESM2_ESM.docx]

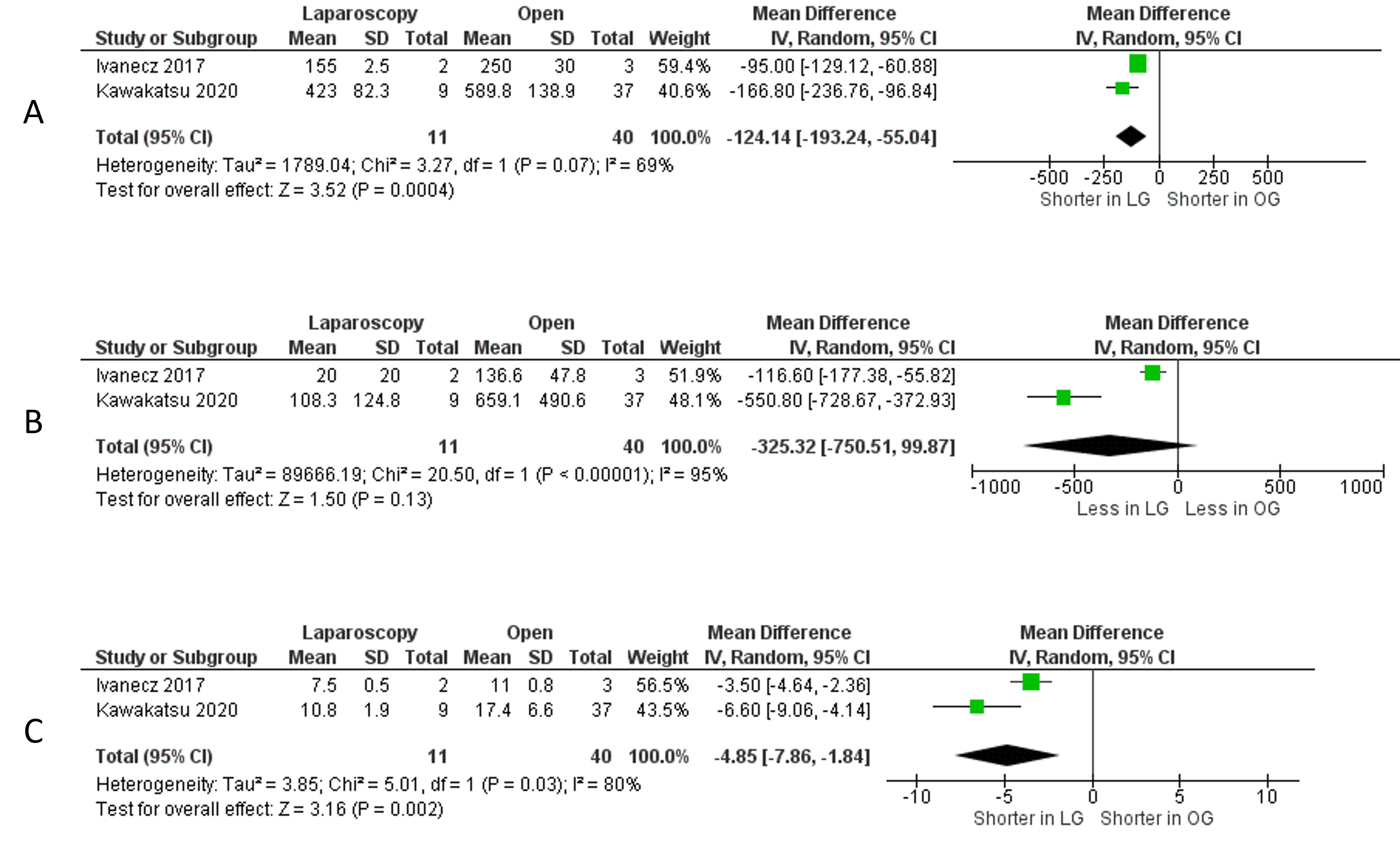


**Supplementary Fig 7:** Subgroup meta-analysis of operative outcomes in patients with primary tumour located in the left colon: (a) operative time; (b) intraoperative blood loss; (c) length of stay

**Legend:** Each study is shown by the point estimate of the odds ratio/mean difference (OR/MD; square proportional to the weight of each study) and 95% confidence interval (CI) for the OR (extending lines); the combined ORs/mean difference and 95% CIs by random effects calculations are shown by diamonds.

(a) Left colon only; LAP versus OPEN and operative time (n=51, p=0.0004; test for heterogeneity Cochran Q: 3.27, df: 1, p=0.07, I^2^: 69%)

(b) Left colon only; LAP versus OPEN and blood loss (n=51, p=0.13; test for heterogeneity Cochran Q: 3.15, df: 2, p<0.00001, I^2^: 95%)

(c) Left colon only; LAP versus OPEN and length of stay (n=51, p=0.002; test for heterogeneity Cochran Q: 5.01, df: 1, p=0.03, I^2^: 80%)
