# Supplementary material for: Laparoscopic versus open resection of primary colorectal cancers and synchronous liver metastasis: a systematic review and meta-analysis
Source: Int J Colorectal Dis. 2023 Apr 5;38(1):90. doi: 10.1007/s00384-023-04375-z (PMC10076361; doi:10.1007/s00384-023-04375-z)
Supplement: Supplementary file 3 — Supplementary file3 (DOCX 622 KB) [file 384_2023_4375_MOESM3_ESM.docx]

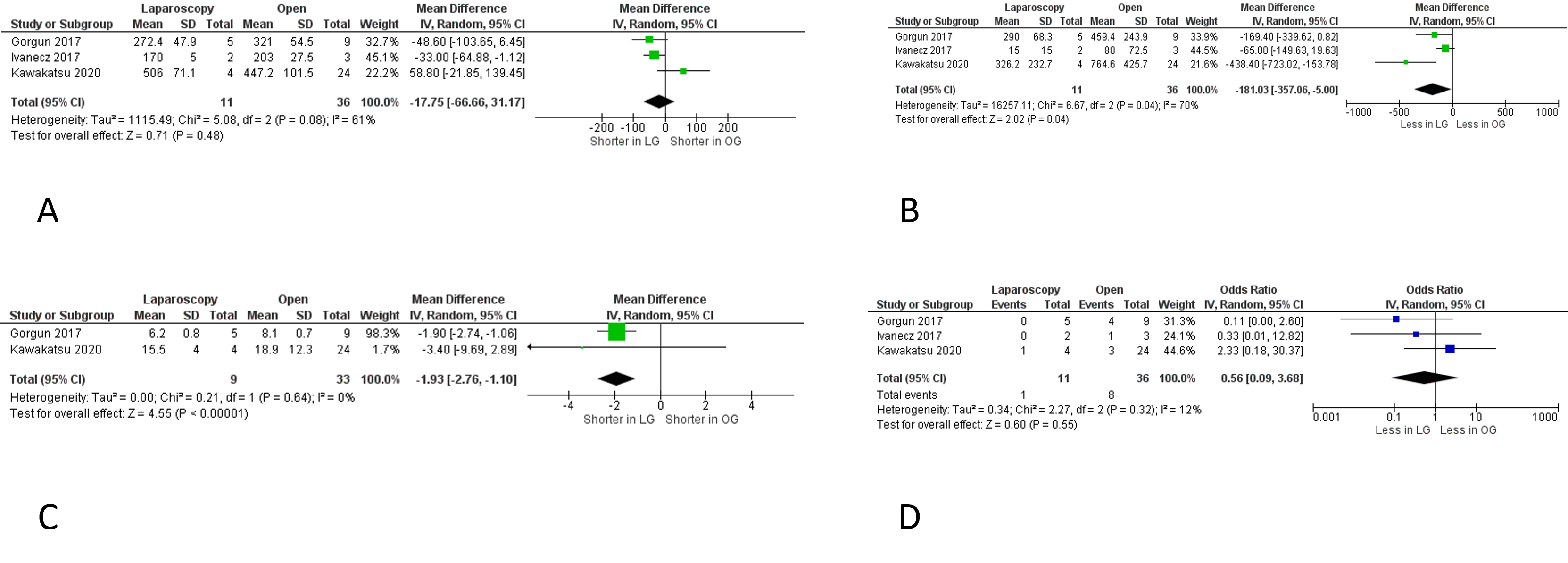


**Supplementary Fig 8:** Subgroup meta-analysis of operative outcomes in patients with primary tumour located in the right colon: (a) operative time; (b) intraoperative blood loss; (c) length of stay; (d) morbidity

**Legend:** Each study is shown by the point estimate of the odds ratio/mean difference (OR/MD; square proportional to the weight of each study) and 95% confidence interval (CI) for the OR (extending lines); the combined ORs/mean difference and 95% CIs by random effects calculations are shown by diamonds.

(a) Right colon only; LAP versus OPEN and operative time (n=47, p=0.48; test for heterogeneity Cochran Q: 5.08 df: 2, p=0.08, I^2^: 61%)

(b) Right colon only; LAP versus OPEN and blood loss (n=47, p=0.04; test for heterogeneity Cochran Q: 6.67, df: 2, p=0.04, I^2^: 70%)

(c) Right colon only; LAP versus OPEN and length of stay (n=42, p<0.00001; test for heterogeneity Cochran Q: 0.21, df: 1, p=0.64, I^2^: 0%)

(d) Right colon only; LAP versus OPEN and morbidity (n=47, p=0.55; test for heterogeneity Cochran Q: 2.27, df: 2, p=0.32, I^2^: 12%)
